# Modification of Acute Stroke Pathway in Korea After the Coronavirus Disease 2019 Outbreak

**DOI:** 10.3389/fneur.2020.597785

**Published:** 2020-11-19

**Authors:** Tae Jung Kim, Beom Joon Kim, Dong-Seok Gwak, Ji Sung Lee, Jun Yup Kim, Keon-Joo Lee, Jung-A Kwon, Dong-Hyun Shim, Yong-Won Kim, Min Kyoung Kang, Eung-Jun Lee, Ki-Woong Nam, Jeonghoon Bae, Kipyoung Jeon, Han-Yeong Jeong, Keun-Hwa Jung, Yang-Ha Hwang, Hee-Joon Bae, Byung-Woo Yoon, Sang-Bae Ko

**Affiliations:** ^1^Department of Neurology, Seoul National University Hospital, Seoul, South Korea; ^2^Department of Critical Care Medicine, Seoul National University Hospital, Seoul, South Korea; ^3^Department of Neurology and Gyunggi Regional Cardiocerebrovascular Center, Seoul National University Bundang Hospital, Seongnam, South Korea; ^4^Department of Neurology, Kyungpook National University Hospital, Daegu, South Korea; ^5^Department of Neurology, School of Medicine, Kyungpook National University, Daegu, South Korea; ^6^Department of Clinical Epidemiology and Biostatistics, Asan Medical Center, University of Ulsan College of Medicine, Seoul, South Korea

**Keywords:** COVID-19, stroke, critical pathway, parameters, modification

## Abstract

**Background:** Since the global pandemic of coronavirus disease 2019 (COVID-19), the process of emergency medical services has been modified to ensure the safety of healthcare professionals as well as patients, possibly leading to a negative impact on the timely delivery of acute stroke care. This study aimed to assess the impact of the COVID-19 pandemic on the acute stroke care processes and outcomes in tertiary COVID-19-dedicated centers in South Korea.

**Methods:** We included 1,213 patients with acute stroke admitted to three centers in three cities (Seoul, Seongnam, and Daegu) through the stroke critical pathway between September 2019 and May 2020 (before and during the COVID-19 pandemic). In all three centers, we collected baseline characteristics and parameters regarding the stroke critical pathway, including the number of admitted patients diagnosed with acute stroke through the stroke critical pathway, door to brain imaging time, door to intravenous recombinant tissue plasminogen activator time, door to groin puncture time, and door to admission time. We performed an interrupted time series analysis to determine the impact of the COVID-19 outbreak on outcomes and critical pathway parameters.

**Results:** Three centers modified the protocol of the stroke critical pathway during the COVID-19 pandemic. There was an immediate decrease in the number of patients admitted with acute ischemic stroke after the outbreak of COVID-19 in Korea, especially in the center of Daegu, an epicenter of the COVID-19 outbreak. However, the number of patients with stroke soon increased to equal that before the Covid-19 outbreak. In several critical pathway parameters, door to imaging time showed a temporary increase, and door to admission was transiently decreased after the COVID-19 outbreak. However, there was no significant effect on the timely trend. Moreover, there was no significant difference in the baseline characteristics and clinical outcomes between the periods before and during the COVID-19 pandemic.

**Conclusion:** This study demonstrated that the COVID-19 outbreak immediately affected the management process. However, it did not have a significant overall impact on the trends of stroke treatment processes and outcomes. The stroke management process should be modified according to changing situations for optimal acute management.

## Introduction

The coronavirus disease 2019 (COVID-19) pandemic caused by the severe acute respiratory syndrome coronavirus 2 has greatly affected healthcare systems worldwide (1). In South Korea, the first case of COVID-19 was identified on January 19, 2020. The patient entered Korea with fever and respiratory symptoms from Wuhan, China. The number of COVID-19 cases increased rapidly since February 17, 2020, at the time of identification of case 31 in Daegu, Kyungbuk, Korea. Therefore, the Korea Disease Control and Prevention Agency raised the alert level and stepped up the social distancing strategy (2, 3). The second wave of COVID-19 spread started in Seoul in August 2020. Currently, the COVID-19 pandemic has led to delays in healthcare services for several medical emergencies, including acute stroke management (1, 4–7). To streamline the process of hyperacute stroke management during the COVID-19 pandemic, several modified recommendations have been reported (6, 8, 9). These guidelines aim to minimize the risk of exposure to COVID-19 for healthcare professionals while maintaining the quality of patient care during the COVID-19 pandemic. However, recent studies showed a reduction in the number of stroke patients requiring reperfusion therapy and those who presented with mild neurological symptoms (10–12). This study aimed to analyze the impact of the COVID-19 outbreak on the changes in acute stroke care processes and outcomes in tertiary COVID-19-dedicated centers in South Korea.

## Methods

### Study Populations

We retrospectively identified consecutive patients in the stroke critical pathway from three tertiary COVID-19-dedicated centers [Center 1 (Seoul National University Hospital) in Seoul, Center 2 (Seoul National University Bundang Hospital) in Seongnam, and Center 3 (Kyungpook National University Hospital) in Daegu] in Korea between September 2019 and May 2020 (before and during the COVID-19 pandemic in Korea on February 17, 2020, identification of case 31 related to a religious group called Shincheonji in Daegu). After reporting case 31, COVID-19 rapidly spread to Daegu/Kyungbuk province and then other areas in Korea. We included 1,213 patients with acute stroke who were admitted to three centers (*n* = 201 in Center 1, *n* = 548 in Center 2, and *n* = 464 in Center 3) through the stroke critical pathway during the period mentioned earlier. This study was approved by the Institutional Review Boards (IRB number H-2007-094-114 in Seoul National University Hospital & Seoul National University Bundang Hospital and 2020-07-055 in Kyungpook National University Hospital).

### Modified Acute Stroke Critical Pathway

Each center had its own protocol for the stroke critical pathway. The initiation criteria for critical pathway in Centers 1 and 2 were based on the last known well time <24 h, and Center 3 focused on the first known abnormal time <24 h. After the declaration of the COVID-19 pandemic, all three centers revised the triage protocol for the stroke critical pathway in the emergency department (ED) to screen for suspected or confirmed COVID-19 infection. COVID-19 diagnostic tests using real-time reverse transcription polymerase chain reaction (sensitivity 92–95% and specificity 94–97%) were performed every 6–12 h (four times per day in Center 1, twice per day in Center 2, and four times per day in Center 3), and the tests took ~5–6 h in three centers. While treating the stoke patients with suspected or confirmed COVID-19, according to the revised stroke critical pathway, a minimum number of healthcare professionals were allowed (one ED physicians, one neurologist, one nurse, one technologist, and/or one emergency medical technician) to limit exposure to COVID-19. Moreover, all institutions were equipped with a computed tomography (CT) scanner at ED, which limited patient transfer time to the CT suite within 5 min. During the COVID-19 pandemic, all patients with suspected stroke were treated according to the modified stroke critical pathway in three centers as described:

#### Criteria 1

Acute stroke patients with laboratory-confirmed COVID-19. The patients are transported to the negative pressure isolation room in each center. After a neurological examination, they undergo brain CT, CT angiography, and CT perfusion in a negative pressure CT suite. Patients eligible for intravenous (IV) thrombolysis are treated according to the standard protocol in the negative pressure isolation room or the negative pressure CT room, depending on the stroke critical pathway process of each center. If a large vessel occlusion is not identified, the patients are transferred to a dedicated special ward or intensive care unit for COVID-19, as needed. If a large vessel occlusion is confirmed, stroke specialists and neuro-interventionalists decide whether to perform endovascular thrombectomy (ERT) based on multimodal CT imaging. ERT is performed in the isolated off-pressure angiography room in each center with a minimum number of healthcare professionals. It is important to ensure level D of personal protective equipment (PPE) is used by all members of staff within the angiography suite. Brain magnetic resonance imaging (MRI) is not performed. All healthcare professionals should wear level D PPE and carried confirmed COVID-19 patients in the isolated negative pressure hood stretcher vehicle along the isolated way (4, 6, 8).

#### Criteria 2

Acute stroke patients with a clinical suspicion of COVID-19 (febrile or respiratory symptoms) or under quarantine due to epidemiological reasons (close contact with a confirmed case or a recent trip to COVID-19-affected regions or abroad within the previous 2 weeks). COVID-19 testing using nasopharynx swabs is performed. Subsequently, the modified stroke critical pathway follows the progress in patients with a confirmed COVID-19. As discussed, patients who needed IV thrombolysis are treated according to the standard protocol in the negative pressure isolation room or negative pressure CT room. Patients with a suspicion of COVID-19 undergo ERT in the isolated off-pressure angiography room with keeping staff to a minimum in the procedure. If IV thrombolysis or ERT is not indicated, the patients are under preemptive isolation until the laboratory diagnosis is finalized. If COVID-19 results come back negative, the patients are transferred to the Stroke Unit; otherwise, they are transferred to the negative-pressure-dedicated special medical ward or intensive care unit for COVID-19, as needed. Moreover, brain magnetic resonance imaging is not performed until the COVID-19 test is negative. All healthcare professionals should wear PPE, including disposable isolation gowns, N95 masks or KF94 (Korea Filter, equivalent to N94) masks, protective goggles, or face shields. Patients should wear a surgical mask during the entire process (4, 6, 8).

#### Criteria 3

Acute stroke patients not diagnosed with COVID-19 and who neither are febrile nor have respiratory symptoms. The patients follow the standard acute stroke management pathway. All staff and patients should wear a surgical face mask throughout the stroke critical pathway (4, 6, 8).

### Clinical Information and Baseline Characteristics

We collected the following parameters regarding acute stroke critical pathway in all three centers: number of admitted patients diagnosed with acute stroke through the stroke critical pathway, number of reperfusion therapy (IV thrombolysis and ERT), door to first brain imaging time, door to recombinant tissue plasminogen activator (rt-PA) time, door to groin puncture time, and door to admission time. In addition, we obtained the number of admitted stroke patients after stroke critical pathway during two periods before the COVID-19 infection from September 2018 and May 2019 to evaluate the seasonal influence on the number of stroke patients. Further, baseline characteristics, vascular risk factors, and pre-stroke functional status of the included patients were obtained by reviewing electronic medical records. Stroke subtypes were classified according to the Trial of Org 10172 in Acute Stroke Treatment criteria: large-artery atherosclerosis, small-vessel occlusion, cardioembolism, or other determined and undetermined subtypes, as previously described (13). Stroke severity was assessed using the National Institutes of Health Stroke Scale (NIHSS) and classified into NIHSS 0–7, NIHSS 8–13, and NIHSS ≥14 (14, 15) in all included patients at admission and discharge. Moreover, functional status at discharge was evaluated using the modified Rankin Scale (mRS). Patients were assigned to either the “favorable outcome” group (mRS score ≤ 2) or “unfavorable outcome” group (mRS score ≥ 3).

### Statistics Analysis

The baseline characteristics of the included patients were presented as the number (%). Further, continuous variables with normal distributions are presented as mean ± standard deviation (SD), whereas variables that were not normally distributed are presented as median value with [interquartile range (IQR)]. Continuous variables were compared using Student's *t*-tests or the Mann–Whitney U test, whereas categorical variables (proportions) were compared using Pearson's χ^2^ tests or Fisher's exact test, as appropriate, to evaluate the impact of the COVID-19 pandemic on changes in the stroke critical pathway. The association between the COVID-19 pandemic and outcomes at discharge (mRS) was analyzed using logistic regression analyses. Covariates with statistically significant differences (*P* < 0.05) by univariate analysis or clinically important factors were adjusted for multivariable analysis. We performed an interrupted time series analysis (ITSA), which implemented a segmented linear regression model, to establish whether there was an association of the COVID-19 explosive outbreak with timely changes in the stroke critical pathway, related parameters, and outcomes (16). We compared the period after February 17, 2020 (the COVID-19 pandemic period) with the pre-COVID-19 pandemic event period (16). A professional medical statistician (J. S. Lee) conducted all statistical analyses using SPSS (Version 25.0; IBM Statistics, Armonk, NY, USA) and SAS 9.4 (SAS Institute, Inc. Cary, NC, USA). Statistical significance was set at *P* < 0.05.

## Results

### Baseline Characteristics of Included Patients

Among the included patients (*n* = 1,213; mean age, 67.8 years; male, 60.3%), 673 (55.5%) patients were admitted before (phase 1) and 540 (44.5%) patients were admitted after (phase 2) the declaration of COVID-19 as a national emergency on February 17, 2020. A between-period comparison of the baseline characteristics in each center revealed no significant differences in the demographic information, vascular risk factors, and pre-stroke functional status, except that hyperlipidemia increased in Center 1 and the proportion of female patients increased in Center 2 during phase 2 (Table 1). Stroke subtypes and mechanisms did not differ between phase 1 and phase 2 in all centers (Table 1). Regarding reperfusion therapy, the proportion of patients with combined IV tPA plus ERT increased in Centers 1 and 3 during phase 2, although there was no significant between-period difference in the proportion of reperfusion therapy in Center 2. Further, more patients presented with fever at ≥37.5°C at the ED in phase 2 in Center 2 and Center 3 (Table 2). During the COVID-19 pandemic period, a total of 46 patients (Criteria 2) were under quarantine in the isolated negative pressure room before admission from three centers. In addition, a total of 181 COVID-19-confirmed patients were treated in three institutions (*n* = 38 in Center 1, *n* = 44 in Center 2, and *n* = 99 in Center 3) until May 2020. However, none was confirmed with COVID-19 among the stroke patients during the stroke critical pathway and after admission in all three centers.

**Table 1:** Baseline characteristics of included patients before and after COVID-19 pandemic in each center.

	**Center 1**			**Center 2**			**Center 3**		
	**Phase 1**	**Phase 2**	***P*-value**	**Phase 1**	**Phase 2**	***P*-value**	**Phase 1**	**Phase 2**	***P*-value**
	**(*n* = 112)**	**(*n* = 89)**		**(*n* = 363)**	**(*n* = 185)**		**(*n* = 345)**	**(*n* = 119)**	
Age, mean (SD)	65.0 (12.4)	66.9 (14.1)	0.309	67.4 (13.8)	68.7 (13.7)	0.312	68.9 (12.9)	67.6 (14.1)	0.362
Male, *n* (%)	62 (55.4)	53 (59.6)	0.551	229 (63.1)	100 (54.1)	0.041	205 (59.4)	82 (68.9)	0.066
Hypertension, *n* (%)	79 (70.5)	63 (70.8)	0.969	250 (68.9)	126 (68.1)	0.856	198 (57.4)	71 (59.7)	0.665
Diabetes mellitus, *n* (%)	37 (33.0)	36 (40.4)	0.278	111 (30.6)	53 (28.6)	0.641	100 (29.0)	40 (33.6)	0.343
Dyslipidemia, *n* (%)	42 (37.5)	49 (55.1)	0.013	170 (46.8)	81 (43.8)	0.498	120 (34.8)	49 (41.2)	0.211
Atrial fibrillation, *n* (%)	19 (17.0)	19 (21.3)	0.430	84 (23.1)	39 (21.1)	0.585	85 (24.6)	23 (19.3)	0.237
Previous stroke/TIA, *n* (%)	13 (11.6)	26 (29.2)	0.002	95 (26.2)	39 (21.1)	0.190	85 (24.6)	29 (24.4)	0.953
Pre-stroke mRS = 0, *n* (%)	80 (71.4)	63 (70.8)	0.921	226 (62.3)	114 (61.6)	0.884	226 (65.5)	80 (67.2)	0.733
Initial NIHSS, median (IQR)	4 (1–9.75)	5 (1.5–12.5)	0.167	4 (2–10)	5 (2–11)	0.175	4 (1–9)	5 (1–9)	0.485
Initial NIHSS, *n* (%)			0.581			0.596			0.456
0–7	77 (68.8)	55 (61.8)		243 (66.9)	120 (64.9)		250 (72.5)	81 (68.1)	
8–13	14 (12.5)	13 (14.6)		58 (16.0)	27 (14.6)		40 (11.6)	19 (16.0)	
≥14	21 (18.8)	21 (23.6)		62 (17.1)	38 (20.5)		55 (15.9)	19 (16.0)	
Stroke subtypes, *n* (%)			0.109			0.081			0.320
Ischemic stroke	87 (78.4)	58 (65.2)		315 (86.8)	147 (79.5)		308 (89.3)	110 (92.4)	
TIA	9 (8.1)	13 (14.6)		19 (5.2)	14 (7.6)		37 (10.7)	9 (7.6)	
Hemorrhagic stroke	15 (13.5)	18 (20.2)		29 (8.0)	24 (13.0)		–	–	
Stroke mechanisms in ischemic stroke, *n* (%)			0.360			0.731			0.248
LAA	15 (17.2)	16 (27.6)		103 (32.7)	52 (35.4)		63 (20.5)	30 (27.3)	
SVO	18 (20.7)	8 (13.8)		48 (15.2)	20 (13.6)		101 (32.8)	31 (28.2)	
CE	21 (24.1)	12 (20.7)		86 (27.3)	33 (22.4)		91 (29.5)	36 (32.7)	
Other determined	16 (18.4)	7 (12.1)		23 (7.3)	11 (7.5)		8 (2.6)	4 (3.6)	
Undetermined	17 (19.5)	15 (25.9)		55 (17.5)	31 (21.1)		45 (14.6)	9 (8.2)	
Discharge NIHSS, median (IQR)	3 (1–5)	4 (1–10)	0.167	4 (1–7)	4 (2–8)	0.056	3 (0–6)	4 (1–8)	0.135

*SD, Standard deviation; CP, critical pathway; mRS, modified Rankin Scale; NIHSS, National Institute of Health Stroke Scale; TIA, transient ischemic attack; IQR, interquartile range;LAA, large artery atherosclerosis; SVO, small vessel occlusion; CE, cardioembolism*.

*Phase 1: before the declaration of COVID-19 as a national emergency on February 17, 2020*.

*Phase 2: after the declaration of COVID-19 as a national emergency on February 17, 2020*.

**Table 2:** Parameters of acute stroke critical pathway before and after COVID-19 pandemic in each center.

	**Center 1**			**Center 2**			**Center 3**		
	**Phase 1**	**Phase 2**	***P*-value**	**Phase 1**	**Phase 2**	***P*-value**	**Phase 1**	**Phase 2**	***P*-value**
	**(*n* = 112)**	**(*n* = 89)**		**(*n* = 363)**	**(*n* = 185)**		**(*n* = 345)**	**(*n* = 119)**	
Number of admitted stroke patients each month through CP, mean (SD)	22.4 (5.3)	29.7 (2.1)	0.069	72.6 (8.1)	61.7 (6.7)	0.098	69.0 (7.4)	39.7 (14.4)	0.008
Fever (> 37.5°C), *n* (%)	2 (1.8)	6 (6.7)	0.142	4 (1.1)	8 (4.3)	0.026	1 (0.3)	6 (5.0)	0.001
Reperfusion therapy, *n* (%)									
IV thrombolysis only	8 (7.1)	9 (10.1)	0.452	22 (6.1)	10 (5.4)	0.757	44 (12.8)	8 (6.7)	0.072
ERT only	7 (6.3)	5 (5.6)	0.851	59 (16.3)	26 (14.1)	0.501	32 (9.3)	13 (10.9)	0.600
Combined IV thrombolysis and ERT	1 (0.9)	7 (7.9)	0.023	21 (5.8)	8 (4.3)	0.470	13 (3.8)	12 (10.1)	0.009
Door to imaging time (min), median (IQR)^†^	25.0 (20.0–33.0)	26.0 (20.0–34.5)	0.835	34.0 (27.0–47.0)	33.0 (24.0–43.0)	0.082	22.5 (18.0–29.0)	19.0 (15.0–24.0)	<0.001
Door to rt-PA time (min), median (IQR)^‡^	50.0 (37.0–71.5)	46.0 (38.8–52.5)	0.388	29.0 (23.0–40.0)	25.0 (23.0–31.0)	0.247	37.5 (30.8–49.3)	46.0 (34.0–58.0)	0.108
Door to groin puncture time (min), median (IQR)	163.5 (92.0–195.8)	110.5 (93.5–133.5)	0.208	73.0 (54.5–101.5)	70.0 (50.3–99.3)	0.490	78.0 (61.0–100.0)	74.0 (61.5–100.0)	0.878
Door to admission time (min), median (IQR)	224.5 (179.8–320.3)	298.0 (185.5–527.5)	0.007	240.5 (181.5–336.3)	195.0 (156.8–250.0)	<0.001	164.0 (113.0–268.0)	125.0 (83.0–255.0)	0.003
Good outcome (mRS 0–2) at discharge, *n* (%)	68 (60.7)	46 (51.6)	0.199	176 (48.5)	74 (40.0)	0.059	218 (63.2)	65 (54.6)	0.099

*SD, Standard deviation; CP, critical pathway; mRS, modified Rankin Scale; NIHSS, National Institute of Health Stroke Scale; TIA, transient ischemic attack; IQR, interquartile range; IV, intravenous; ERT, endovascular reperfusion therapy; rt-PA, recombinant tissue plasminogen activator*.

*Phase 1: before the declaration of COVID-19 as a national emergency on February 17, 2020*.

*Phase 2: after the declaration of COVID-19 as a national emergency on February 17, 2020*.

*Door to imaging time^†^ Door to rt-PA time^†^: Center 2 is a regional comprehensive stroke center with a high volume of patients transfer from nearby primary stroke centers. Therefore, if a patient arrives at Center 2 within tPA time window with brain CT, which was undergone at primary stroke centers, then the patient may be administered with intravenous tPA. This is why door to tPA time is shorter than door to imaging time in Center 2*.

### Parameters of Stroke Critical Pathway Before and During the Coronavirus Disease 2019 Pandemic Period

**Figure 1:**
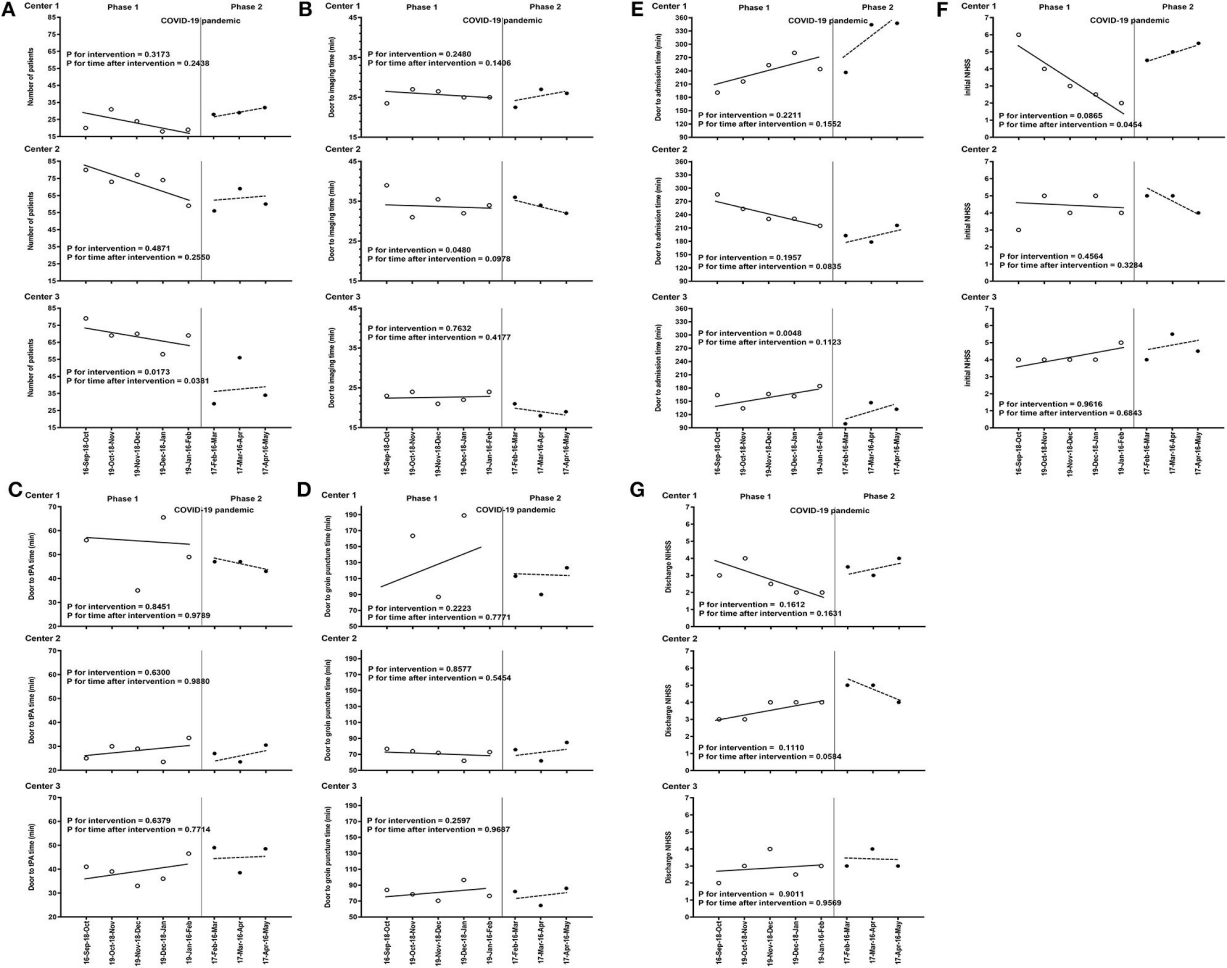
Trends in monthly parameters of the stroke critical pathway between September 2019 and May 2020. Dashed lines represent the COVID-19 pandemic since the confirmed 31 cases related to a religious group called Shincheonji in Daegu. **(A)** Trends in the number of admitted patients in each center. **(B)** Trends in the door to imaging time in each center. **(C)** Trends in the door to rt-PA time in each center. **(D)** Trends in the door to groin puncture time in each center. **(E)** Trends in the door to admission time in each center. **(F)** Trends in initial NIHSS of patients with stroke in each center. **(G)** Trends in discharge NIHSS of patients with stroke in each center. COVID-19, coronavirus disease; rt-PA, tissue plasminogen activator; NIHSS, National Institutes of Health Stroke Scale. Phase 1: before the declaration of COVID-19 as a national emergency on February 17, 2020. Phase 2: after the declaration of COVID-19 as a national emergency on February 17, 2020.

In Center 3 at Daegu, fewer stroke patients were admitted in phase 2 compared with phase 1 (39.7 ± 14.4 vs. 69.0 ± 7.4, *P* = 0.008 in Table 2). Breakpoint analysis revealed that there was a significant decrease in the number of patients with stroke in the stroke critical pathway immediately after the COVID-19 pandemic (*P* for intervention = 0.0173 in Figure 1A). Although there was an increasing trend in the number of patients with stroke, fewer patients were admitted in phase 2 compared with phase 1 (*P* for time after intervention = 0.0381 in Figure 1A). However, in Center 1 and Center 2, there was no significant difference in the trend of the number of admitted patients (Figure 1A). When analyzing the trend during the same time frame in 2019, the number of admitted stroke patients was significantly decreased in February, associated with the shortest month of the year; however, there was an increase in the number of patients with stroke in other months. During the COVID-19 pandemic period, the numbers of admission were persistently lower in 2020 compared with that in 2019. Furthermore, the trend of admitted stroke patients was different compared with the same time frame in 2019 (Supplementary Figure 1). In Center 3, the median door to imaging time during the COVID-19 pandemic was significantly shorter than that before the COVID-19 period in univariate analysis (*P* <0.001 in Table 2). However, in ITSA, there was no significant difference in the trend of the door to imaging time after the COVID-19 outbreak (Figure 1B). In Center 2, the door to imaging time was transiently higher after the COVID-19 outbreak, but the time trend was not significant (*P* = 0.097, Figure 1B). The door to tPA time was shorter in Center 2 compared with other centers because all tPA-treated patients (*n* = 61) in Center 2 were performed in CT room during the study period, although there was no significant change in the time trend of each center during the COVID-19 pandemic. Among under quarantine patients (*n* = 46), four patients were treated with tPA according to Criteria 2. The door to tPA time was longer compared with without quarantine patients (*n* = 50), according to Criteria 3 [56.5 (IQR 34.8–64.8) vs. 36.0 (IQR 27.5–47.8) min, *P* = 0.090], although it was not statistically significant result. Compared with before the COVID-19 period, univariate analysis showed that the door to admission time after the COVID-19 period was significantly longer in Center 1 and shorter in Center 2 and Center 3. In Center 3, there was a transient decrease in the door to admission time; however, it showed an increasing time trend after the COVID-19 outbreak (Figure 1E). In Centers 1 and 2, the COVID-19 pandemic effect on the time trend was not significant after ITSA. Moreover, there was no significant change in parameters related to reperfusion therapy between before and after the COVID-19 pandemic in all three centers (Figures 1C,D). The initial stroke severity was similar between the two periods (Table 1). Although there was an increasing trend in the initial NIHSS (*P* for time after intervention = 0.0454 in Figure 1F) in Center 1, the NIHSS and mRS scores of all centers were nearly similar at discharge after the COVID-19 pandemic period compared with before the COVID-19 period (Tables 1, 2, Figures 1F,G). In addition, the COVID-19 pandemic was not associated with discharge outcomes after adjusting for the relevant confounding variables (Supplementary Table 1) in the three centers. Moreover, when comparing the initial and discharge stroke severity according to the under quarantine during the COVID-19 pandemic period, there was no significant difference between under quarantine group and without quarantine group [initial NIHSS 6 (IQR, 2–19.5) vs. 5.0 (IQR 2–11), *P* = 0.098, discharge NIHSS 4 (IQR 2–22) vs. 4 (IQR 1–8), *P* = 0.117, respectively].

## Discussion

We found that the monthly number of admitted patients with acute stroke in the stroke critical pathway decreased immediately after the declaration of national emergency of COVID-19, especially at Center 3, which was located at the epicenter of the COVID-19 outbreak. However, there was an inconsistent and nonsignificant impact of the COVID pandemic event on the time trend of the number of patients with stroke in three centers. During the COVID-19 pandemic period, there was a transient change in the parameters of the stroke critical pathway, including door to imaging time and door to admission time. However, there was no between-period difference in the hyperacute treatment process and short-term post-stroke outcomes. Differences in stroke critical pathway parameters, including door to imaging time and door to tPA time, are related to regional differences and own stroke critical pathways of the three centers, which is not directly associated with the COVID-19 outbreak.

Previous studies have reported a reduced number of patients with a minor stroke or transient ischemic attack, as well as reperfusion therapy. Further, there could have been delayed reperfusion therapy, onset to door, and door to treatment times after the COVID-19 outbreak (4, 10, 11, 17–19). In our study, there was a temporary reduction in the number of patients with stroke in the critical pathway during the COVID-19 pandemic, which was consistent with previous studies (10–12). However, the trend subsequently recovered to the normal state-observed before the COVID-19 outbreak. In Center 3 at Daegu, which comprised 31 cases related to the Shincheonji religious group, there was a temporary decrease in the number of patients during the COVID-19 pandemic period compared with that before the COVID-19 pandemic period. However, ITSA revealed an increasing trend in the number of admitted patients after a critical pathway with the passing of time. Furthermore, the long-term COVID-19 impact on reperfusion therapy remains unclear. Regarding the door to admission time, the temporary reduction in the door to admission time in Center 3 was associated with the transient number of patients in the stroke critical pathway and stroke unit availability immediately after the COVID-19 explosive outbreak. However, there was an increasing time trend of the door to admission time in Center 3, as the time trend of the number of patients was increased after the COVID-19 explosive outbreak. During the stage after the COVID-19 outbreak, each center attempted to establish a modified protocol strategy for acute stroke management that reflected regional characteristics. Therefore, the early-stage protocol is associated with the transient change of stroke critical pathway parameters in each center. We found that the overall characteristics and outcomes of admitted patients with stroke after the critical pathway remained stable. The strategy of hyperacute management did not significantly change during the post-COVID-19 pandemic period. In this study, four patients with clinical suspicion of COVID-19 were treated with tPA in the isolated negative pressure room or negative pressure CT room. The quarantine lengthened the door to tPA time in the quarantine group without statistical significance [56.5 (IQR 34.8–64.8) vs. 36.0 (IQR 27.5–47.8) min, *P* = 0.090], consistent with previous studies (19, 20). Their delays were attributed to when applying the PPE and additional time taken to isolate the patient. However, the initial severity [NIHSS 5.5 (IQR, 3.5–23.3) in under quarantine vs. 9 (IQR, 5–14.5) without quarantine, *P* = 0.690] and discharge outcome [NIHSS 3 (IQR, 0.5–32.5) in under quarantine vs. 4 (IQR, 1–8) in without quarantine, *P* = 0.811] were similar in two groups among all tPA-treated patients. Moreover, the COVID-19 explosive outbreak had a temporary impact on the number of admitted patients with stroke after the critical pathway and parameters associated with the critical pathway. Thus, there was no significant effect on the trends for the critical pathway parameters in this study. Moreover, the admitted COVID-19-confirmed patients were transferred through the secure pathway in each center. Therefore, the impact of the COVID-19-confirmed patients was not significant in the stroke critical pathway of each center.

These findings could be attributed to several possible explanations. Many countries, including Korea, have implemented strategies for controlling the COVID-19 spread, including social distancing; shutting down schools, churches, gyms, and bars; wearing of masks, washing of hands, and activity restrictions (10, 20). The COVID-19 is a contact-transmissible infectious disease thought to spread throughout the population via direct individual–individual contact; moreover, it is yet to have effective antiviral medications and vaccines. Consequently, some patients with stroke may refrain from visiting emergency treatment at hospitals for fear of infection, which could have attributed to the decreased number of admitted patients with stroke during the early COVID-19 periods (3, 16, 21). Most stroke centers have modified and optimized the triage protocols for acute stroke management for the prevention of COVID-19 spread with respect to regional characteristics (1, 8, 9, 22). In Korea, the spread of emerging infectious diseases was slowed down followed by a flattening of the epidemic curve after consistent implementation of government policy and strategy (10, 22). In our study, each center maintained optimism for providing effective stroke therapies after establishing modified triage protocols after the COVID-19 outbreak. Consequently, there was a transient COVID-19 impact on the critical pathway of patients with stroke, which remained nonsignificant after epidemic curve flattening and establishing the modified stroke critical pathway in each center.

This study has several limitations. First, this was a triple-center retrospective study. Therefore, there remains a possibility of selection bias, and caution should be applied when generalizing these findings to the clinical field. Second, the study period was insufficient for analyzing the impact of the COVID-19 infection breakpoint on the stroke critical pathway using ITSA. Moreover, the sample size was small to show statistical significance. Therefore, although our findings were nonsignificant in the time trend, they should be interpreted with caution. Third, the association between the severity of the COVID-19-infected patients and the admission, treatment, and outcome in patients after stroke critical pathway was not evaluated in this study because there was no admitted stroke patient with COVID-19 infection during this study period. Fourth, the change of variables related to ERT was not analyzed after the COVID-19 outbreak among the three centers. Fifth, this study might not represent the country and regions with different stroke care protocols and geographical specificities. The result of this study could be related to acute stroke treatment guidelines in South Korea. Therefore, the generalization of results could be limited in low- and middle-income developing countries because of their lack of acute stroke management systems. Thus, there is a need for these findings to be confirmed in other centers and populations. Further, our findings could be limited to community hospitals and small centers.

In conclusion, this study demonstrated that the COVID-19 explosive outbreak had an immediate acute effect on the hyperacute stroke management process within a short period. However, there was an insignificant overall impact of the COVID-19 pandemic on the trends of the stroke treatment process and outcomes. Stroke management is a dynamic process that is modifiable with changing situations. Implementation of a modified stroke pathway compatible with infection control in each stroke center ensured that the efficiency of the overall stroke management process was retained. There is a need for further large-scale studies to confirm the true relationship between the COVID-19 explosive outbreak and the long-term effect on the stroke management process.

## Data Availability Statement

The original contributions presented in the study are included in the article/Supplementary Materials, further inquiries can be directed to the corresponding author/s.

## Ethics Statement

The studies involving human participants were reviewed and approved by Institutional Review Boards (IRB number H-2007-094-114 in Seoul National University Hospital & Seoul National University Bundang Hospital and 2020-07-055 in Kyungpook National University Hospital). Written informed consent for participation was not required for this study in accordance with the national legislation and the institutional requirements.

## Author Contributions

S-BK, B-WY, H-JB, Y-HH, and K-HJ contributed to the study concept and design. TJK, BJK, D-SG, JYK, K-JL, J-AK, D-HS, Y-WK, MKK, E-JL, K-WN, JB, KJ, and H-YJ contributed to data collection. TJK and JSL contributed to data analysis. TJK and S-BK drafted the manuscript.

## Conflict of Interest

The authors declare that the research was conducted in the absence of any commercial or financial relationships that could be construed as a potential conflict of interest.
