# Supplementary material for: Modification of Acute Stroke Pathway in Korea After the Coronavirus Disease 2019 Outbreak
Source: Front Neurol. 2020 Nov 19;11:597785. doi: 10.3389/fneur.2020.597785 (PMC7710988; doi:10.3389/fneur.2020.597785)
Supplement: Supplementary file 1 [file Data_Sheet_1.docx]

**Supplementary Figure 1. The number of admitted patients following the stroke critical pathway during two periods (from September 2018 to May 2019 versus from September 2019 to May 2020)**

**
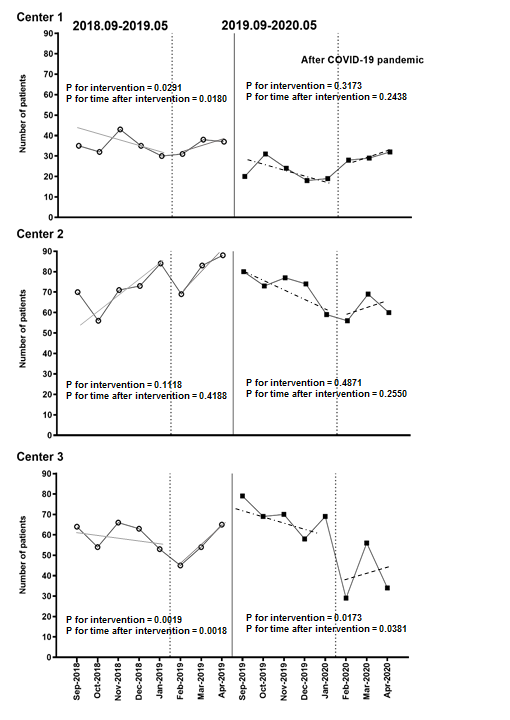
**

Dashed lines between September 2019 and May 2020 represent the COVID-19 pandemic since the confirmed 31 cases related to a religious group called Shincheonji in Daegu

**Supplementary Table 1.** **Multivariable analyses of the relationship between the COVID-19 pandemic and outcome at discharge in admitted patients with acute stroke after stroke critical pathway from three centers**

|  | **Crude odds ratio** | **95% CI** | ***P* value** | **Adjusted odds ratio** | **95% CI** | ***P* value** |
| --- | --- | --- | --- | --- | --- | --- |
| **Center 1** |  |  |  |  |  |  |
| **Age** | 1.038 | 1.014-1.062 | 0.002 | 1.035 | 0.997-1.074 | 0.071 |
| **Initial NIHSS** | 1.496 | 1.329-1.683 | <0.001 | 1.527 | 1.344-1.735 | <0.001 |
| **The COVID-19 pandemic** | 1.445 | 0.823-2.536 | 0.200 | 1.133 | 0.467-2.749 | 0.782 |
| **Center 2** |  |  |  |  |  |  |
| **Age** | 1.038 | 1.025-1.052 | <0.001 | 1.063 | 1.039-1.087 | <0.001 |
| **Initial NIHSS** | 1.419 | 1.327-1.517 | <0.001 | 1.440 | 1.329-1.560 | <0.001 |
| **The COVID-19 pandemic** | 0.319 | 0.100-1.018 | 0.054 | 1.576 | 0.919-2.703 | 0.098 |
| **Center 3** |  |  |  |  |  |  |
| **Age** | 1.057 | 1.039-1.075 | <0.001 | 1.050 | 1.026-1.075 | <0.001 |
| **Initial NIHSS** | 1.344 | 1.266-1.427 | <0.001 | 1.362 | 1.269-1.462 | <0.001 |
| **The COVID-19 pandemic** | 0.312 | 0.105-0.928 | 0.036 | 1.568 | 0.863-2.849 | 0.140 |

NIHSS: National Institute of Health Stroke Scale

Outcome: poor outcome mRS 3-6 at discharge

Adjusting for age, initial NIHSS, door to imaging time, door to admission time, and COVID-19 pandemic event
